# Report of the Diseases of Edinburgh for February, 1809

**Published:** 1809-04

**Authors:** John Roberton


### *Report of the Diseases of Edinburgh for February*, 1809. By John Roberton, M. D.

In the beginning of this month the weather was soft, and, especially, during the night, we had showers of rain. This was immediately succeeded by heavy falls of rain and snow, which continued almost incessantly for several days. The state of the air during this period, particularly after sun-set, was excessively unpleasant, being thick and very ill-calculated for the comfort of those labouring under asthmatic complaints. About the end of the first third of the month the weather became dry, and the air consequently much more wholesome. The frost now was so severe, that the thermometer was sometimes below the freezing point. This state of the weather, with the addition of slight falls of snow, and some very heavy gales of wind, continued till about the middle of the month, when the weather again became soft. The gales of wind, however, still continued for a week more, and, from about this period till the end of the month, the state of the weather was variable, and accompanied by occasional gales of wind.

During the first half of the month in particular, the barometer was in general low, though it occasionally varied ried considerably; but, during the latter part of the month, it generally stood high.

The thermometer also varied considerably: in the earlier parts of the month it was sometimes as high as 40, or from that to 50 degrees, at other times considerably below the freezing point. In general, however, during the latter part of the month, it stood at from 40 to 50 degrees, and sometimes even above this.

The complaints which principally prevailed were inflammatory affections of different parts, particularly of the lungs and abdominal viscera, which now seem beginning rather to relax in their general severity, typhus fevers of a very bad kind, catarrh, cynanche tonsillaris, and measles.

Although I have observed above, that inflammatory complaints have somewhat abated in their severity, yet some cases occur in practice of the very worst kind, from which, independently of the most active treatment, very little relief has been obtained, at least for a considerable length of time, and, in some cases, the violent symptoms did not in the smallest degree relax, consequently the patients died. Some, however, who, from the strength of their constitution, did escape after being affected in this violent manner, were often afterward seized with dropsy, which at length terminated fatally.

Typhus fever has, as before observed, occurred, particularly among the lower orders of society, where it perpetually originates, and is often diffused over the whole city. It is curious, that although this fever most commonly prevails in greatest severity in London, for instance, only during the spring, or more particularly in the summer months, we find that in Edinburgh it is equally severe at all seasons. I do not recollect to have seen this fever here, when uncombined with any other disease, of a more malignant nature at any one time of the year than at another. The only difference that seems, in this respect, to exist respecting it is, that during the summer months and about the autumn, its ravages are more extensive than in the winter; but, from its frequently appearing in all its severity, even during the coldest weather, we see that, at least here, the cold has no effect in rendering it milder.

Catarrh and cynanche tonsillaris have, either separately or combined, been diseases of the most extensive nature. For the cure of the first, the avoiding of sudden alternations from extremes of heat and cold, with the occasional use of saline laxative medicines, have generally removed it in a few days. The same attention to medicines and regimen has also been found necessary for the cure of cynanche, with the addition, in some cases, of gargles, and a blister applied over the throat.

The measles have neither been so general, nor so fatal, as they were last year in this place. Indeed, I believe scarcely any cases of them have terminated fatally.

About the end of the month, in consequence of the circulation of some reports respecting the existence of hydrophobia among us, the magistrates issued a proclamation prohibiting the appearance of dogs in the streets, except under proper restrictions. This, from the supposed dreadful nature of the disease, has occasioned much alarm in the mind of the inhabitants, but it does not appear that any of them have suffered from its effects.

What is the reason that we know so little of this hydrophobia, which every one talks of, and so few understand? The scattered remarks in insulated cases, said to be of this disease, give us but an imperfect idea of its nature. On the contrary, I believe, they have sometimes been the source of mistakes, having led to the supposition of hydrophobia being present, when a very different disease in reality existed.

The incorrect notion which the world in general entertain of this malady is well known : it seems more like a tale of superstition, worked up in proportion to the briliancy of the relator's imagination, than any real disease, the nature of which may, by a little attention, be well known, or the entire prevention or cure of which might be effected on scientific principles.

However philanthropic the disposition of many may be, yet, considering the well known neglect, if not positive opposition, which is too often all the reward given for exertions in the public service, they are little to be blamed for suppressing the most useful truths, particularly when their time and abilities may be more beneficially employed among those to whom they are immediately known. On this account, I believe many such truths and truly benevolent schemes for the public good, have often been allowed to remain with the proposer, till partly from the above causes, and partly from the short span of human life, he has been prevented from ever making their real nature appear, and consequently with himself they have sunk into oblivion.

Although these or similar observations may be applied, in a great measure, to the causes which have prevented us from obtaining a more perfect knowledge of the disease menmentioned above, I have no doubt that we may, by a little attention to certain points, in some degree remove them.

The present brutal plan, at least in this place, of employing men, void of all feeling, to hover about the streets at all hours with bludgings, for the purpose of butchering any dog that may come in their way, is, in my opinion, very far from answering any good end; but putting entirely out of the question the immoral effect which it must have, both on those employed in it, and on those who witness it, on the contrary, is calculated to produce much mischief. These men are allowed a small sum of money for the head of every dog they can produce, as a proof of their success in that kind of traffic; and I have no doubt that, under the influence of such an offer, very unfair means must be used for the purpose of enticing animals from houses, for the express purpose of murdering them. But, independently of all this, the horrible acts of cruelty which every inhabitant is obliged, while walking the streets, to witness, and thus to have his feelings perpetually outraged or annihilated, cannot be expressed by language. Half murdered dogs, who have by their superior strength escaped destruction, are by no means uncommon spectacles, and in this state, irritated by the stabs and blows which they had just received, they are certainly very apt to fly at the first person they meet. Thus, instead of such measures proving a public benefit, they are positively a public calamity, and I have no doubt that when they are duly considered, all proclamations, in whatever part of the country, tending to encourage such acts of brutality, will be entirely laid aside. On the contrary, I should propose that some of the principal inhabitants of every town and city, form themselves into a sort of committee. Let them order Essays or Observations to be delivered on the subject of Hydrophobia, and let these be impartially examined; impartially, I say, for this is too seldom done; and let a reward, such as a medal, or other mark of honour, be given for the best and most satisfactory scientific account of that terrible disease, and an equally scientific plan for its prevention or removal.

I may observe, that I can have no motive but the welfare and safety of my fellow mortals, in making the above observations and proposal. I am desirous of knowing more of the disease, not of standing candidate in the above scheme. In short, I am desirous of being enabled to obviate the excruciating sufferings which it may be my misfortune to witness.

4, *St. James's Street.*

